# Living Preference Modifies the Associations of Living Arrangements With Loneliness Among Community-Dwelling Older Adults

**DOI:** 10.3389/fpubh.2021.794141

**Published:** 2022-01-21

**Authors:** Kai Wei, Junjie Yang, Bixi Yang, Lijuan Jiang, Jiangling Jiang, Xinyi Cao, Chunbo Li

**Affiliations:** ^1^Shanghai Key Laboratory of Psychotic Disorders, Shanghai Mental Health Center, Shanghai Jiao Tong University School of Medicine, Shanghai, China; ^2^Shanghai Institute of Traditional Chinese Medicine for Mental Health, Shanghai, China; ^3^Clinical Neurocognitive Research Center, Shanghai Mental Health Center, Shanghai Jiao Tong University School of Medicine, Shanghai, China; ^4^Institute of Psychology and Behavioral Science, Shanghai Jiao Tong University, Shanghai, China; ^5^CAS Center for Excellence in Brain Science and Intelligence Technology (CEBSIT), Chinese Academy of Science, Shanghai, China; ^6^Brain Science and Technology Research Center, Shanghai Jiao Tong University, Shanghai, China

**Keywords:** living preferences, living arrangements, loneliness, effect modification, cohort analysis

## Abstract

**Objectives:**

Living arrangement has been reported to have a significant influence on feelings of loneliness in older adults, but their living preferences may confound the association. This study aimed to investigate whether the associations of living arrangements with loneliness differ in community-dwelling older adults according to different living preferences.

**Methods:**

In the 2008/2009 (baseline) and 2011/2012 (follow-up) waves of the Chinese Longitudinal Healthy Longevity Survey, living arrangements [living with children mainly (LWC), living with spouse only (LWS), and living alone (LA)], living preferences [preferring living with children (PreLWC) and preferring living alone/only with spouse (PreLA)], and feelings of loneliness were assessed. The effect modifications of living preferences in the associations of living arrangements with loneliness were estimated using logistic regression models, and corresponding odds ratios (*OR*s) were calculated.

**Results:**

Living preferences significantly modified the associations of living arrangements with loneliness at baseline (*p* for interaction = 0.009 for LWS and = 0.015 for LA). Compared with LWC, LWS was protective for loneliness only in the PreLA older adults at baseline (*OR* = 0.53, 95% *CI* = 0.45–0.64, *p* < 0.001), and LA was significantly associated with loneliness especially in the PreLWC older adults, compared with their PreLA counterparts (at baseline, *OR*s = 2.89 *vs*. 2.15; at follow-up, *OR*s = 1.68 *vs*. 1.51).

**Conclusion:**

Living preference modifies the associations of living arrangements with loneliness, and those who prefer living with children but live alone are more likely to feel lonely. It is recommended that living preferences should be considered when managing loneliness in community-dwelling older adults.

## Introduction

As filial piety of the Confucius culture prevails in China, co-residence is valued as the most desirable living arrangement for community-dwelling older adults (1). However, with the development of our society and increasing preferences for individual privacy and independence, recognition of older adults on living alone, another kind of living arrangement, is changing. Living alone in older adults could be an initiative choice for socioeconomic advantaged older adults with younger age and better health status, who prefer a lifestyle of more freedom and privacy (2), or an involuntary choice for older adults who suffer from certain socioeconomic disadvantages (such as, being childless or in poverty) and need to live alone (3). Living alone is often accompanied by a decreased level of family/social support and healthcare utilization, leading to social isolation and life challenges for older adults (4, 5). Living alone older adults have been found more likely to be lonely compared with their counterparts (3, 6), and loneliness is a major source of suffering among older adults (7). Previous studies have demonstrated that loneliness increased the risk of developing dementia among older adults especially in men (8), and was associated with mental disorders, such as depression, physical decline, and increased risk of death (9, 10).

Although closely associated, living alone and loneliness have different definitions: the former is an objective measure of one's living arrangements and is associated with social isolation, while the latter is a subjective emotional experience of one's personal relationships (11). Loneliness can be explained as the lack of “meaningful” social relationships (12), or the discrepancy between desired and actual relationships of an individual, either in quantity or quality (10). *Deficit* and *cognitive* explanations have been proposed for loneliness. With regard to the first, which focuses on the situational factors that cause loneliness, people need social contacts to avoid loneliness, and the lack of such contacts directly results in feelings of loneliness (13), but situational factors do not entirely explain the differences between lonely and not lonely individuals (14). As a subjective experience, loneliness cannot be fully understood without taking preferences or aspirations of people for their social contacts into account (13). According to the *cognitive* view, loneliness arises when actual social relationships do not match those that are desired (13). When this discrepancy exceeds a threshold, then the feelings of loneliness emerge. The *symbolic interactionism* emphasizes that the situation, individual, and individual–situation interaction play important roles during aging (15). Based on this, the *social environment model* is designed and proposed three key factors: the expectation on norm derived from specific situation, the ability of individual communicative competence, and the consistence on subjective evaluation between ability and expectation in specific situation. The degree of harmony of the three factors determines happiness and quality of life in older adults (15).

According to the above theories, loneliness is tied to the magnitude of social network of an individual, but mainly depends on how that individual subjectively perceives those relationships and how satisfied an individual is with the types of support received from those relationships (16, 17). This means that two older adults with similar social resources may have different feelings of loneliness (17), among which the subjective living preferences would play a modifying role but have been seldomly investigated. For older adults, living in a preferred arrangement could help them to adjust their needs and expectations and adapt to their living environments, so as to spend their late-life happily (14, 18).

To investigate the modifying effects of living preferences in the associations of living arrangements with loneliness in community-dwelling older adults, our study used the cross-sectional and longitudinal data of the population-based Chinese Longitudinal Healthy Longevity Survey (CLHLS) in the 2008/2009 and 2011/2012 waves and included 13,364 community-dwelling older adults for the analyses. We examined the sociodemographic, socioeconomic, physical, and cognitive factors related to living arrangements and living preferences, assessed the associations of living arrangements with loneliness, and determined whether living preference modifies these associations among community-dwelling older adults aged 65 years or above in China.

## Methods

### Study Design and Participants

The CLHLS is an ongoing, prospective cohort study of community-dwelling Chinese older adults (19, 20). It covers the majority of the provinces in China and aims to investigate the factors associated with healthy longevity of Chinese. Started in 1998, the follow-ups have been conducted every 2–3 years. To reduce attrition, new participants are continually enrolled as death and lost-to-follow-up are inevitable. Trained interviewers with a structured questionnaire conduct the survey from door to door. Written informed consent was obtained from all participants and/or their proxy respondents, and the study was approved by the Research Ethics Committee of Peking University (IRB00001052-13074). All methods were performed in accordance with the relevant guidelines and regulations.

In the 2008/2009 wave (baseline), 16,954 older adults were initially interviewed, the number of which was the most among different waves. We excluded 391 participants younger than 65 years, 508 participants with living preferences “institution/don't know,” 308 participants living in an institution [as they were much older (93.1 ± 9.1 years), and institution living was different from community-dwelling], and 2,383 participants without definite status of loneliness [who were much older (96.6 ± 7.7 years) and mostly cognitively impaired (96%)], and finally included 13,364 community-dwelling older adults for analyses, among who 54.9% (7,342/13,364) survived, 29.3% (3,920/13,364) died, and 15.7% (2,102/13,364) were lost in the 3-year follow-up (the 2011/2012 wave). The baseline characteristics of older adults according to follow-up status were shown in Supplementary Table S1.

### Measurements

We used the data of living preferences and living arrangements at baseline, and assessed the associations of living arrangements with loneliness in the total sample and stratified by living preferences, at baseline and 3-year follow-up.

#### Assessment of Living Preference and Living Arrangement

Living preference was assessed *via* the question “What kind of living arrangement do you like best?,” with answers “living alone (or only with spouse) regardless of proximity to children” (10.2%), “living alone (or only with spouse) with close proximity to children” (31.9%), and “living with children” (58.0%). The former two answers were combined as “preferring living alone (PreLA)” and the third as “preferring living with children (PreLWC).”

Living arrangement was assessed using the question “Who do you live with?” with responses “living with family (including housemaid)” and “living alone (LA).” We further defined “living with spouse only (LWS)” if “living with family (including house maid)” older adults had the answer to the question “How many people are living with you?” as “1” and answer to “Relationship between you and 1st person you live with currently” as “spouse,” otherwise “living with children mainly (LWC)” was defined. Answers to “Relationship between you and 1st person you live with currently” including “spouse (37.2%),” “child/spouse of child (55.7%),” “grandchild/spouse of grandchild (3.6%),” and “others (3.5%).”

#### Loneliness

Loneliness was assessed *via* the question “Do you feel lonely or isolated?” with answers “always,” “often,” “sometimes,” “seldom,” and “never,” which is demonstrated to be feasible for the loneliness assessment by previous studies (21, 22). For the purpose of statistical analysis, we recoded the responses into dichotomous data as follows: “always,” “often,” and “sometimes,” were defined as “lonely (32.7%),” “seldom,” and “never” as “not lonely (67.3%).”

#### Covariates

Measures of sociodemographic characteristics at baseline included age, gender, race (Han Chinese or minority), SDW, Single/Separated/Divorced/Widowed, residence (rural or city/town), occupation (non-professional or professional), education (<1 year or ≥1 year), body mass index (BMI), smoking, alcohol drinking, socioeconomic status (such as, sufficient financial support, economic independence, adequate medical service, and public medical payment), and dietary habits (such as fruit and vegetable eating and tea drinking). Social/leisure activity score was calculated in the way same as a previous study (23), with a higher score representing a higher frequency of social and leisure activities. Physical exercise was assessed *via* the question “Do you take exercise or not at present?” with answers ‘yes’ or ‘no.’ Self-reported health was assessed *via* the question “How do you rate your health status?” with answers ‘bad’ and ‘very bad’ defined as “poor self-reported health.” Interviewer-rated health was assessed by interviewers, with “moderately ill” and “very ill” defined as “poor interviewer-rated health.” Comorbidity was assessed *via* whether suffering from 24 common chronic diseases listed in the questionnaire (e.g., heart diseases, stroke, diabetes, hypertension, cancers, cataracts, and Parkinson's disease). Serious illness in the past 2 years was defined as “illness that causes hospitalization or being bedridden all the year around.” In addition, hearing and visual ability were assessed. Frailty Index (FI) score was calculated *via* the number of reported deficits divided by the total number of included deficits (ranging from 0 to 1), with a higher value indicating severer frailty. The continuous FI score was classified into non-frailty (FI score ≤ 0.21) and frailty (FI score > 0.21) following previous reports (24, 25). The Chinese version of the MMSE was used to measure cognitive function of older adults, and education-adjusted cognitive impairment was defined according to the same criteria in previous studies (26).

### Data Analysis

Categorical variables were presented as numbers (percentages), and continuous variables were presented as means (SD). Differences in the distribution of categorical variables among groups were tested by χ^2^ test. For continuous variables, the *F*-test or Kruskall–Wallis test was used for comparison between different groups. Logistic regression models were performed to assess the associations of living arrangements with loneliness in the total sample and stratified by living preferences, and calculate the corresponding odds ratios (*OR*s) and 95% *CI*s. To test whether living preference was an effective modifier, interaction terms between living preferences and living arrangements for prevalent loneliness were assessed in logistic regression models, adjusted for baseline values of age, gender, race, residence, occupation, education, BMI, smoking, alcohol drinking, socioeconomic status, dietary habits, social/leisure activity score, physical exercise, poor self-rated health, poor interviewer-rated health, comorbidities (≥2), serious illness in the past 2 years, hearing problem, visual impairment, cognitive impairment, and frailty. Marital status was not adjusted as it overlaps with living arrangement. *OR* estimates for prevalent loneliness were adjusted for the same set of confounders cited above. As many variables changed from 2008/2009 to 2011/2012, interaction terms and *OR* estimates for incident loneliness were adjusted for age, gender, race, education, occupation, and changes in other variables from 2008/2009 to 2011/2012. The multicollinearity of the covariates adjusted in the above regression models was assessed by calculating their variance inflation factor (VIF) values (<10 indicating no collinearity). The acceptable level of significance was set as two-sided *p* < 0.05. Stata version 14.0 (StataCorp LP, College Station, TX, USA) was used for data analysis.

## Results

### Baseline Characteristics by Living Arrangements and Living Preferences

As shown in Table 1, some of the factors associated with LA and PreLA were similar. In general, LA and PreLA older adults were more likely to be younger, Han-Chinese, currently smoking, and drinking alcohol; more of them took physical exercise, and fewer had poor interviewer-rated health, hearing problem, visual impairment, frailty, and cognitive impairment, compared with their LWC and PreLWC counterparts, respectively. Compared with the LWC ones, LA older adults were more likely to be SDW, live in rural, prefer living alone, have relatively worse socioeconomic status, and eat fewer fruits and vegetables; fewer of them had serious illness in the past 2 years, and more had poor self-rated health and prevalent and incident loneliness. PreLA older adults were more likely to be male, married, live in city/town, have professional occupation, better education, higher BMI, better socioeconomic status and dietary habits, higher social/leisure activity score, and fewer of them had prevalent loneliness, compared with those who preferred LWC.

**Table 1:** Baseline characteristics by living arrangements and living preferences.

**Characteristics**	**Total Sample**	**LWC**	**LWS**	**LA**	***P***	**PreLWC**	**PreLA**	***P***
	**(*N* = 13364)**	**8414 (63.0)**	**2807 (21.0)**	**2143 (16.0)**		**7746 (58.0)**	**5618 (42.0)**	
**Sociodemographic**								
Age (years)^*^	85.7 (11.1)	88.2 (11.1)	78.1 (8.6)	85.8 (9.6)	**<0.001**	88.6 (10.8)	81.7 (10.3)	**<0.001**
Gender (female)	7329 (54.8)	5102 (60.6)	953 (34.0)	1274 (59.5)	**<0.001**	4752 (61.4)	2577 (45.9)	**<0.001**
Race (minority)	875 (6.6)	658 (7.8)	101 (3.6)	116 (5.4)	**<0.001**	661 (8.5)	214 (3.8)	**<0.001**
Marital status (SDW)	8788 (65.8)	6651 (79.1)	18 (0.6)	2119 (98.9)	**<0.001**	6184 (79.8)	2604 (46.4)	**<0.001**
Residence (rural)	7983 (59.7)	4968 (59.0)	1667 (59.4)	1348 (62.9)	**0.005**	4739 (61.2)	3244 (57.7)	**<0.001**
Occupation (professional)	1012 (7.6)	542 (6.5)	352 (12.6)	118 (5.5)	**<0.001**	448 (5.8)	564 (10.1)	**<0.001**
Education (≥1 year)	5366 (40.2)	3021 (36.0)	1608 (57.3)	737 (34.5)	**<0.001**	2674 (34.6)	2692 (48.0)	**<0.001**
BMI (kg/m^2^)^*^	20.4 (3.5)	20.2 (3.4)	21.4 (3.7)	20.0 (3.3)	**<0.001**	20.0 (3.4)	21.1 (3.6)	**<0.001**
Current smoker	2427 (18.2)	1319 (15.7)	722 (25.7)	386 (18.0)	**<0.001**	1176 (15.2)	1251 (22.3)	**<0.001**
Current alcohol drinker	2396 (17.9)	1354 (16.1)	678 (24.2)	364 (17.0)	**<0.001**	1201 (15.5)	1195 (21.3)	**<0.001**
Prefer living alone	5618 (42.0)	1625 (19.3)	2350 (83.7)	1643 (76.7)	**<0.001**	-	-	-
**Socioeconomic status**								
Sufficient financial support	10564 (79.1)	6731 (80.0)	2222 (79.2)	1611 (75.2)	**<0.001**	6045 (78.0)	4519 (80.4)	**0.001**
Economic independence	3686 (27.6)	1885 (22.4)	1306 (46.5)	495 (23.1)	**<0.001**	1608 (20.8)	2078 (37.0)	**<0.001**
Adequate medical service	12463 (93.3)	7903 (93.9)	2638 (94.0)	1922 (89.7)	**<0.001**	7184 (92.7)	5279 (94.0)	**0.005**
Public medical payment	1804 (13.5)	1030 (12.2)	542 (19.3)	232 (10.8)	**<0.001**	842 (10.9)	962 (17.1)	**<0.001**
**Dietary habits**								
Fruit eating	5340 (40.0)	3413 (40.6)	1252 (44.6)	675 (31.5)	**<0.001**	3002 (38.8)	2338 (41.6)	**0.001**
Vegetable eating	11891 (89.0)	7469 (88.8)	2584 (92.1)	1838 (85.8)	**<0.001**	6863 (88.6)	5028 (89.5)	0.106
Tea drinking	5529 (41.4)	3297 (39.2)	1374 (49.0)	858 (40.0)	**<0.001**	2962 (38.3)	2567 (45.7)	**<0.001**
**Physical and cognitive health status**							
Social/leisure activity score (point)^*^	3.5 (3.1)	3.2 (3.0)	4.9 (3.3)	3.1 (2.8)	**<0.001**	3.1 (2.9)	4.2 (3.2)	**<0.001**
Physical exercise	4007 (30.0)	2259 (26.9)	1083 (38.6)	665 (31.0)	**<0.001**	1988 (25.7)	2019 (35.9)	**<0.001**
Poor self-reported health	2090 (15.7)	1232 (14.7)	495 (17.6)	363 (17.0)	**<0.001**	1238 (16.0)	852 (15.2)	0.193
Poor interviewer-rated health	1845 (13.8)	1293 (15.4)	307 (10.9)	245 (11.4)	**<0.001**	1228 (15.9)	617 (11.0)	**<0.001**
Comorbidities (≥2)	6178 (46.2)	3809 (45.3)	1442 (51.4)	927 (43.3)	**<0.001**	3555 (45.9)	2623 (46.7)	0.357
Serious illness in the past 2 years	2201 (16.5)	1350 (16.1)	558 (19.9)	293 (13.7)	**<0.001**	1256 (16.2)	945 (16.8)	0.349
Hearing problem	2193 (16.4)	1758 (20.9)	165 (5.9)	270 (12.6)	**<0.001**	1625 (21.0)	568 (10.1)	**<0.001**
Visual impairment	2125 (15.9)	1588 (18.9)	212 (7.6)	325 (15.2)	**<0.001**	1515 (19.6)	610 (11.0)	**<0.001**
Frailty	3141 (23.5)	2546 (30.3)	270 (9.6)	325 (15.2)	**<0.001**	2381 (30.7)	760 (13.5)	**<0.001**
Cognitive impairment	2532 (19.0)	1987 (23.7)	211 (7.5)	334 (15.6)	**<0.001**	1845 (23.9)	687 (12.3)	**<0.001**
Prevalent loneliness	4367 (32.7)	2783 (33.1)	503 (17.9)	1081 (50.4)	**<0.001**	2750 (35.5)	1617 (28.8)	**<0.001**
Incident loneliness^#^	1238 (25.3)	674 (24.7)	361 (22.5)	203 (36.1)	**<0.001**	612 (25.7)	626 (24.9)	0.529

*Data presented as n (%) or mean (SD). LWC, living with children mainly; LWS, living with spouse only; LA, living alone; PreLWC, preferring living with children; PreLA, preferring living alone/only with spouse; SDW, Single/Separated/Divorced/Widowed*.

* *Kruskall-Wallis test was used, data presented as mean (SD); for other characteristics, χ2 test was used and data presented as n (%), similarly hereinafter*.

# *For 4897 older adults who were not lonely at baseline and had feelings of loneliness at follow-up. The bold values in the P columns indicating significant differences*.

It is worth noting that the LWS older adults had the best status compared with those in other living arrangements: they were youngest, most likely to have professional occupation and ≥1 year education, had highest BMI, best socioeconomic status and dietary habits, most social/leisure activities and physical exercise, and fewest of them had poor interviewer-rated health, hearing problem, visual impairment, frailty, cognitive impairment, and prevalent and incident loneliness. Besides, most of them were male, Han-Chinese, currently smoking and drinking alcohol, and most preferred LA, although most of them had ≥2 comorbidities and serious illness in the past 2 years. In addition, we found that both LWS and LA older adults tended to underestimate their health status, indicated by the fact that fewer had poor interviewer-rated health but more had poor self-rated health.

### Baseline Characteristics by Combinations of Living Preferences and Living Arrangements

In our study, 71% of the PreLA older adults achieved their preference (such as LWS: 41.8% or 2,350/5,618, LA: 29.3%, or 1,643/5,618), and 88% (6,789/7,746) of the PreLWC ones achieved their preference. As shown in Table 2, PreLWC and LWC older adults (accounted for 50.8% in the total sample) were oldest, mostly female, and minority; fewest of them were currently smoking, economic independent, drinking tea, or took physical exercise, and most of them had poor interviewer-rated health, hearing problem, visual impairment, frailty, and cognitive impairment. Most of those who preferred LWC but LWS (accounted for 3.4% in the total sample) lived in rural, had ≥2 comorbidities, and serious illness in the past 2 years. Older adults who preferred LWC but LA accounted for 3.7% in the total sample, and showed characteristics, such as fewest professional occupation and ≥1 year education, lowest BMI, fewest current alcohol drinking, worst socioeconomic status, fewest fruit and vegetable eating, lowest social/leisure activity score, fewest ≥2 comorbidities, and most poor self-rated health, and prevalent and incident loneliness. Older adults who preferred LA but LWC (accounted for 12.2% in the total sample) were most likely to live in city/town, have relatively better socioeconomic status, and were least likely to have poor self-reported health. Those who preferred LA and LWS (accounted for 17.6% in the total sample) were youngest, and most were male, Han-Chinese, currently smoking and drinking alcohol; most of them had professional occupation and ≥1 year education, highest BMI, best socioeconomic status and dietary habits, most social/leisure activities and physical exercise, and fewest had poor interviewer-rated health, hearing problem, visual impairment, frailty, cognitive impairment, and prevalent and incident loneliness. Fewest of the older adults who PreLA and LA (accounted for 12.3% in the total sample) were married, and fewest had serious illness in the past 2 years.

**Table 2:** Baseline characteristics stratified by the combinations of living preferences and living arrangements.

**Characteristics**	**PreLWC and LWC**	**PreLWC but LWS**	**PreLWC but LA**	**PreLA but LWC**	**PreLA and LWS**	**PreLA and LA**	***P***
	**6789 (50.8)**	**457 (3.4)**	**500 (3.7)**	**1625 (12.2)**	**2350 (17.6)**	**1643 (12.3)**	
**Sociodemographic**							
Age (years)	89.4 (10.7)	79.3 (9.1)	86.5 (9.1)	83.1 (11.4)	77.9 (8.5)	85.6 (9.7)	**<0.001**
Gender (female)	4263 (62.8)	177 (38.7)	312 (62.4)	839 (51.6)	776 (33.0)	962 (58.6)	**<0.001**
Race (minority)	602 (8.9)	24 (5.3)	35 (7.0)	56 (3.5)	77 (3.3)	81 (4.9)	**<0.001**
Marital status (SDW)	5683 (83.7)	7 (1.5)	494 (98.8)	968 (59.6)	11 (0.5)	1625 (98.9)	**<0.001**
Residence (rural)	4115 (60.6)	302 (66.1)	322 (64.4)	853 (52.5)	1365 (58.1)	1026 (62.5)	**<0.001**
Occupation (professional)	381 (5.6)	43 (9.4)	24 (4.8)	161 (9.9)	309 (13.2)	94 (5.7)	**<0.001**
Education (≥1 year)	2260 (33.4)	253 (55.4)	161 (32.3)	761 (47.0)	1355 (57.7)	576 (35.1)	**<0.001**
BMI (kg/m^2^)	20.0 (3.4)	20.7 (3.3)	19.2 (2.9)	21.1 (3.6)	21.6 (3.7)	20.3 (3.4)	**<0.001**
Current smoker	981 (14.5)	111 (24.3)	84 (16.8)	338 (20.8)	611 (26.0)	302 (18.4)	**<0.001**
Current alcohol drinker	1052 (15.5)	86 (18.8)	63 (12.6)	302 (18.6)	592 (25.2)	301 (18.3)	**<0.001**
**Socioeconomic status**							
Sufficient financial support	5409 (79.7)	310 (67.8)	326 (65.2)	1322 (81.4)	1912 (81.4)	1285 (78.2)	**<0.001**
Economic independence	1314 (19.4)	195 (42.7)	99 (19.8)	571 (35.1)	1111 (47.3)	396 (24.1)	**<0.001**
Adequate medical service	6359 (93.7)	406 (88.8)	419 (83.8)	1544 (95.0)	2232 (95.0)	1503 (91.5)	**<0.001**
Public medical payment	737 (10.9)	59 (12.9)	46 (9.2)	293 (18.0)	483 (20.6)	186 (11.3)	**<0.001**
**Dietary habits**							
Fruit eating	2715 (40.0)	164 (35.9)	123 (24.6)	698 (43.0)	1088 (46.3)	552 (33.6)	**<0.001**
Vegetable eating	6049 (89.1)	399 (87.3)	415 (83.0)	1420 (87.4)	2185 (93.0)	1423 (86.6)	**<0.001**
Tea drinking	2562 (37.8)	192 (42.0)	208 (41.6)	735 (45.2)	1182 (50.3)	650 (39.6)	**<0.001**
**Physical and cognitive health status**						
Social/leisure activity score (point)	3.0 (2.9)	4.4 (3.2)	2.6 (2.5)	4.0 (3.2)	5.0 (3.2)	3.2 (2.9)	**<0.001**
Physical exercise	1705 (25.1)	147 (32.2)	136 (27.2)	554 (34.1)	936 (39.8)	529 (32.2)	**<0.001**
Poor self-reported health	1024 (15.1)	100 (21.9)	114 (22.9)	208 (12.8)	395 (16.8)	249 (15.2)	**<0.001**
Poor interviewer-rated health	1080 (15.9)	71 (15.5)	77 (15.4)	213 (13.1)	236 (10.0)	168 (10.2)	**<0.001**
Comorbidities (≥2)	3083 (45.4)	256 (56.0)	216 (43.2)	726 (44.7)	1186 (50.5)	711 (43.3)	**<0.001**
Serious illness in the past 2 years	1079 (15.9)	102 (22.3)	75 (15.0)	271 (16.7)	456 (19.4)	218 (13.3)	**<0.001**
Hearing problem	1528 (22.5)	32 (7.0)	65 (13.0)	230 (14.2)	133 (5.7)	205 (12.5)	**<0.001**
Visual impairment	1376 (20.3)	41 (9.0)	98 (19.6)	212 (13.1)	171 (7.3)	227 (13.8)	**<0.001**
Frailty	2227 (32.8)	59 (12.9)	95 (19.0)	319 (19.6)	211 (9.0)	230 (14.0)	**<0.001**
Cognitive impairment	1716 (25.3)	39 (8.5)	90 (18.1)	271 (16.7)	172 (7.3)	244 (14.9)	**<0.001**
Prevalent loneliness	2319 (34.2)	122 (26.7)	309 (61.8)	464 (28.6)	381 (16.2)	772 (47.0)	**<0.001**
Incident loneliness^#^	519 (25.2)	57 (24.9)	36 (38.3)	155 (23.2)	304 (22.0)	167 (35.7)	**<0.001**

*Data presented as n (%) or mean (SD). LWC, living with children mainly; LWS, live with spouse only; LA, living alone; PreLWC, preferring living with children; PreLA, preferring living alone/only with spouse; SDW, Separated/Divorced/Widowed*.

# *For 4897 older adults who were not lonely at baseline and had feelings of loneliness at follow-up. The bold values in the P columns indicating significant differences*.

### Effect Modifications of Living Arrangements on Loneliness by Living Preferences

As shown in Table 3, compared with the LWC older adults, LWS was protective for prevalent loneliness at baseline (*OR* = 0.63, 95% *CI* = 0.55–0.72, *p* < 0.001), but no longer protective for incident loneliness in the 3-year follow-up (*OR* = 0.97, 95% *CI* = 0.79–1.20, *p* = 0.806), and the LA older adults were significantly more likely to be lonely at both baseline (*OR* = 2.43, 95% *CI* = 2.14–2.76, *p* < 0.001) and follow-up (*OR* = 1.66, 95% *CI* = 1.30–2.12, *p* < 0.001), although with decreased *OR* from baseline to follow-up.

**Table 3:** Modification of the effect of living arrangement on loneliness by living preferences.

	**LWC**		**LWS**		**LA**		**LWS**	**LA**
	**N With/Without Loneliness**	**Odds Ratio (95% CI)**	**N With/Without Loneliness**	**Odds Ratio (95% CI)**	**N With/Without Loneliness**	**Odds Ratio (95% CI)**	**Odds Ratio (95% CI) for the Association Between Living Arrangement and Loneliness Within Each Stratum of Living Preferences**	
**Cross-sectional Analyses^a^**								
Total Sample^#^	2783/5631	1.0 (reference)	503/2304	0.63 (0.55–0.72) *P* < 0.001	1081/1062	2.43 (2.14–2.76) *P* < 0.001	-	-
PreLWC	2319/4470	1.0 (reference)	122/335	0.79 (0.62-1.01) *P* = 0.064	309/191	2.92 (2.35-3.62) *P* < 0.001	0.81 (0.63-1.04) *P* = 0.103	2.89 (2.33-3.60) *P* < 0.001
PreLA	464/1161	0.98 (0.86–1.13) *P* = 0.828	381/1969	0.53 (0.46-0.61) *P* < 0.001	772/871	2.05 (1.81-2.33) *P* < 0.001	0.53 (0.45-0.64) *P* < 0.001	2.15 (1.82-2.54) *P* < 0.001
**Longitudinal Analyses^b^**							
Total Sample ^#^	674/2053	1.0 (reference)	361/1247	0.97 (0.79–1.20) *P* = 0.806	203/359	1.66 (1.30 - 2.12) *P* < 0.001	-	-
PreLWC	519/1541	1.0 (reference)	57/172	1.21 (0.84–1.75) *P* = 0.303	36/58	1.70 (1.05–2.75) *P* = 0.032	1.16 (0.79–1.69) *P* = 0.450	1.68 (1.03–2.74) *P* = 0.038
PreLA	155/512	1.22 (0.96–1.56) *P* = 0.101	304/1075	1.08 (0.89–1.31) *P* = 0.447	167/301	1.89 (1.48–2.41) *P* < 0.001	0.87 (0.67–1.13) *P* = 0.304	1.51 (1.12–2.06) *P* = 0.008

*LWC, living with children mainly; LWS, live with spouse only; LA, living alone; PreLWC, preferring living with children; PreLA, preferring living alone/only with spouse*.

a *Measure of effect modification on multiplicative scale: LWS: OR (95% CI) = 0.68 (0.50–0.91), P = 0.009; LA: OR (95% CI) = 0.71 (0.54–0.94), P = 0.015. Adjusted for age, gender, race, residence, occupation, education, BMI, smoking, alcohol drinking, socioeconomic status, dietary habits, social/leisure activity score, physical exercise, poor self-rated health, poor interviewer-rated health, comorbidities (≥2), serious illness in the past 2 years, hearing problem, visual impairment, cognitive impairment, and frailty*.

b *Measure of effect modification on multiplicative scale: LWS: OR (95% CI) = 0.73 (0.47–1.13), P = 0.159; LA: OR (95% CI) = 0.91 (0.52–1.61), P = 0.749. For 4897 older adults who were not lonely at baseline and had feelings of loneliness at follow-up. Adjusted for age, gender, race, education, occupation, and changes in residence, BMI, smoking, alcohol drinking, socioeconomic status, dietary habits, social/leisure activity score, physical exercise, self-rated health, interviewer-rated health, comorbidity number, serious illness in the past 2 years, hearing problem, visual impairment, cognitive impairment, and frailty from 2008/2009 to 2011/2012*.

# *Living preference was also adjusted*.

When stratified by living preferences, compared with the LWC older adults, LWS was protective for prevalent loneliness only in PreLA older adults at baseline (*OR* = 0.53, 95% *CI* = 0.45–0.64, *p* < 0.001), but not the PreLWC ones (*OR* = 0.81, 95% *CI* = 0.63–1.04, *p* = 0.103); the *OR* between LA and prevalent loneliness was decreased by PreLA (*OR*=2.15, 95% *CI* = 1.82–2.54, *p* < 0.001), while further increased by PreLWC (*OR* = 2.89, 95% *CI* = 2.33–3.60, *p* < 0.001). Although the same trend was found in the analysis of follow-up, only LA was significantly associated with the increased risk of incident loneliness in PreLWC and PreLA older adults (*OR* = 1.68, 95% *CI* = 1.03–2.74, *p* = 0.038; *OR* = 1.51, 95% *CI* = 1.12–2.06, *p* = 0.008, respectively). Living preference significantly modified the associations of living arrangements with prevalent loneliness (*p* for interaction = 0.009 for LWS, = 0.015 for LA) but not incident loneliness (*p* for interaction = 0.159 for LWS, = 0.749 for LA).

## Discussion

In terms of elderly care, familism, especially filial piety, remains a predominant norm in the intergenerational relationship in China (27), thus living alone is generally not a preferred option for most Chinese older adults. Living with spouse and/or children could facilitate material, emotional, and other instrumental supports to older adults, and promote communications with family members; by contrast, living alone is often accompanied by a decreased family/social support and social isolation (4, 5). However, with the development of our society and increasing preferences for individual privacy and independence, recognition of older adults on living alone is changing. Some studies have found that LWC could increase dependence, family obligations, losses of privacy, and self-determination, thus speeding up age-related loss of physical ability (2, 28), while LA older adults have more free time and less family obligations and other constraints, which may help them to be more socially active (29). Additionally, LA has been demonstrated to be a conditioned choice of a set of critical factors among older adults: those who have higher education, financial independence or higher income, available housing, and good health status could afford to live alone (3, 30).

According to the *deficit* and *cognitive* theories, although living alone may cause loneliness in older adults, loneliness cannot be fully understood without considering their preferences for social relationships. Only when actual social relationships do not match those that are desired, will loneliness emerge (13, 14). Fundamentally, emotion is the interaction between an individual and a situation that evokes emotion, but not the simple summation of them. Thus, individual-situation interaction plays an important role in emotional regulation. Situation selection, the most prospective emotion regulation strategy from *emotional regulation process model* (31), emphasizes putting the individual in a situation that is more likely to produce satisfactory emotions, or avoiding situations that may produce unsatisfactory emotions. Older adults can better predict the emotional arousal and experience brought by the current situation, and adopt situation selection strategy more in emotional regulation (32). Therefore, living preferences of older adults, which are usually culture-based, may play a modifying role in the associations of living arrangements with loneliness.

In our study, LWS was the most favorable living arrangement for the married older adults (61%, 2,789/4,576), consistent with previous studies (27, 33). Nearly all LA older adults were SDW (99%) and widowhood accounted for 96% in the SDW at baseline, indicating that widowhood was the major cause for them to live alone. Besides, SDW older adults may choose to live with their children, as 76% (6,651/8,788) of the SDW older adults were LWC and 70% (6,184/8,788) of them preferred LWC in our study. It is reasonable for us to consider that LA was most likely a kind of personal choice for some older adults, as 77% (1,643/2,143) of the LA older adults preferred LA (much higher than 42% in the total sample). For some LWC older adults, however, LWC may be a kind of reluctant actions, as 19% (1,625/8,414) of the LWC older adults preferred LA.

The voluntary LWS/LA accounted for 29.9% in the total sample (such as, 17.6% PreLA and LWS, and 12.3% PreLA and LA), and the involuntary LWS/LA accounted for only 7.1% in the total sample (such as, 3.4% PreLWC but LWS and 3.7% PreLWC but LA). Regardless of their living preferences, compared with the LWC older adults, the LWS/LA ones were more likely to live in rural, and the rural-urban migration of their adult children in China may be an important reason, leaving them as “empty nesters” (3). Childlessness, however, was not the potential reason in our study, as very few of them had no alive child (data not shown). Additionally, it is worth noting that the involuntary LWS/LA rural older adults had relatively worse physical and socioeconomic status. Although the proportion of these older adults was not high in the total sample, they need more attention and social service, which is currently in shortage.

Living with spouse only/living alone and PreLA needed some preconditions. Older adults who were younger, male, Han-Chinese, had better education, more physical exercise, and better physical and cognitive functions were more likely to LWS/LA and PreLA. Although both LWS and LA older adults were more likely to prefer LA and underestimate their own health status, the LWS ones were also more likely to live in city/town, have better socioeconomic status and dietary habits, serious illness in the past 2 years, and less likely to be lonely at both baseline and follow-up, while the LA older adults were on the contrary. More of the PreLA older adults lived in city/town with better socioeconomic status and dietary habits, more social/leisure activities, and less prevalent loneliness. When considering the combinations of living preferences and living arrangements, generally, the PreLWC and LWC older adults were oldest, had least physical exercise, and worst physical and cognitive functions; most of the PreLWC but LWS ones lived in rural and had chronic/serious physical diseases; the PreLWC but LA ones were more likely to live in rural, had worst socioeconomic status, dietary habits, fewest social/leisure activities, and most prevalent and incident loneliness; most of the PreLA but LWC ones lived in city/town, had relatively better socioeconomic status, and best self-rated health; the PreLA and LWS older adults were youngest, had best socioeconomic status and dietary habits, most social/leisure activities and physical exercise, best interviewer-rated health, best physical and cognitive functions, and least prevalent and incident loneliness; the PreLA and LA ones were least likely to have physical diseases.

Living with spouse only was not consistently protective for loneliness at follow-up, while the LA older adults had decreased *OR* of loneliness from baseline to follow-up, no matter in the total sample or stratified by living preferences. The former may be caused by the change of marital status in late life: about 17% of the LWS older adults experienced marital change from being married to SDW in our study, regardless of their living preferences. Compared with consistent SDW (from baseline to follow-up) older adults, those who experienced marital change from being married to SDW (widowhood accounted for 96% in the SDW at follow-up) were significantly more likely to have incident loneliness (adjusted *OR* = 1.48, 95% *CI* = 1.10–1.99, *p* = 0.010). The death of a spouse signifies the loss of a significant attachment figure that likely provided the most meaningful and intimate source of social support and may lead to loneliness (34). The latter may be due to the situation that they got used to LA and successfully readjusted their expectations about social support and social relationships according to their actual situation at follow-up (17). Previous studies have reported that loneliness induced by widowhood decreased for most older adults in a similar manner, regardless of how much loneliness they initially reported (35).

Living preference played a modifying role in the associations of living arrangements with loneliness. Only voluntary LWS was protective for loneliness at baseline, and involuntary LA older adults had higher *OR*s of loneliness than the voluntary LA ones at both baseline and follow-up. When stratifying the status of loneliness, the involuntary LA older adults had much higher *OR*s of loneliness (“sometimes,” “often,” and “always”) than the voluntary LA ones at baseline (Supplementary Table S2). In addition, we noticed that involuntary LWC (the PreLA but LWC) was marginally associated with loneliness at follow-up (*OR* = 1.22, *p* = 0.101), compared with older adults who preferred LWC and LWC, indicating that living in a non-preferred arrangement may increase the risk of loneliness in older adults over time, even for those relatively younger older adults with good socioeconomic and health status.

In China, the “family standard” and “responsibility ethics” were the core in the culture of elderly care. The former emphasizes that family is prior to individual, and the latter has more implications: older adults would give to their children (including grandchildren) without expecting any favor in return, be tolerant on unfulfillment of children in filial piety, try their best to reduce children's repayment in financial support, life care and spiritual comfort, as well as be self-reliant when they lost the ability to give (18). In rural community, under the dominance of the “family standard” culture, the value of persons' life is their responsibility to the family. Rural-living older adults are keen on comparing with others in the life achievements of individuals or families, which are centered on responsibility of individual to the family and the prosperity of the family. Elderly care seems to be their personal affair, which does not involve the prosperity of the family, nor does it involve their life achievements. Therefore, elderly care may be neglected in rural community, and such behavioral norms and mentality have been recognized by the community. However, in rural community with relatively more developed economy, some older adults have accepted the value of individualism to some extent while adhering to the collectivism value of the “family standard” culture, indicating that elderly care is becoming diverse along with the social transformation (18). Although the “responsibility ethics” caused self-reliance in older adults is prevalent in China, it does not mean we should neglect the responsibilities of their adult children and our society. From original intention of older adults, the choice of “responsibility ethics” has cultural and voluntary reasons, but they are also likely to be forced to lower their living standards to reduce repayment of children, which need special attention when investigating the situation of elderly care. In our study, the LWS/LA older adults who preferred LWC were more likely to live in rural and had relatively worse physical or socioeconomic status. According to the “family standard” and “responsibility ethics,” these older adults really needed but did not get elderly care from their adult children. The detailed reasons of this phenomenon should be investigated, and corresponding measures should be adopted for this population. Meanwhile, some older adults, who live in city/town and have better socioeconomic and health status, may prefer individualism more than collectivism, but have to live with and offer help to their children involuntarily. For this population, their late-life living preference should be respected and corresponding social service should be applied by our society, so as to help them to live in a preferred arrangement.

An important contribution of this study is that we find the modifying role of living preference in the associations of living arrangements with loneliness. However, some limitations still exist in our study. First, PreLA included PreLWS in our study, but we could not separate the latter from the former, which may cause some confusion in understanding our results. We found that 53.6% of the PreLA older adults were married, and they may prefer LWS, which needs further investigation to clarify. Second, living preferences and living arrangements were both dynamic, but we only considered the modifying effects of baseline living preferences in the associations of baseline living arrangements with prevalent and incident loneliness, which may cause some bias. However, when analyzing these modifying effects at follow-up, changes in some sociodemographic, socioeconomic, physical, and cognitive characteristics were adjusted in our analyses, which could guarantee the reliability of our results to some extent. In the future research, detailed living preferences of older adults, the kinds of violations to living preferences as well as the specific causes, and factors associated with dynamic changes of living preferences and living arrangements should be investigated to better manage the loneliness and reduce corresponding adverse health outcomes in older adults.

In conclusion, living preference plays a modifying role in the associations of living arrangements with loneliness. Living alone is more closely associated with loneliness in older adults who prefer living with children. It is recommended that living preferences should be considered when managing loneliness in community-dwelling older adults.

## Data Availability Statement

The datasets presented in this study can be found in online repositories. The names of the repository/repositories and accession number(s) can be found below: https://opendata.pku.edu.cn/dataset.xhtml?persistentId=doi:10.18170/DVN/WBO7LK.

## Ethics Statement

The studies involving human participants were reviewed and approved by Research Ethics Committee of Peking University. The patients/participants provided their written informed consent to participate in this study.

## Author Contributions

CL and XC supervised the data analysis and revised the manuscript. KW planned the study, reviewed the literature, performed the data analysis, interpreted the results, and drafted the manuscript. JY, BY, LJ, and JJ reviewed the literature, interpreted the results, and reviewed the manuscript. All authors read and approved the final manuscript.

## Funding

This work was supported by the National Key Research and Development Program of China (grant numbers 2018YFC2001605, 2020YFC2003000); the Shanghai Clinical Research Center for Mental Health (grant number 19MC1911100); the Key Program of Clinical Research Center in Shanghai Mental Health Center (grant number CRC2017ZD01); the Shanghai Municipal Science and Technology Major Project (grant number 2018SHZDZX03) and ZJLab.

## Conflict of Interest

The authors declare that the research was conducted in the absence of any commercial or financial relationships that could be construed as a potential conflict of interest.

## Publisher's Note

All claims expressed in this article are solely those of the authors and do not necessarily represent those of their affiliated organizations, or those of the publisher, the editors and the reviewers. Any product that may be evaluated in this article, or claim that may be made by its manufacturer, is not guaranteed or endorsed by the publisher.
