# Supplementary material for: Living Preference Modifies the Associations of Living Arrangements With Loneliness Among Community-Dwelling Older Adults
Source: Front Public Health. 2022 Jan 21;9:794141. doi: 10.3389/fpubh.2021.794141 (PMC8814323; doi:10.3389/fpubh.2021.794141)
Supplement: Supplementary file 1 [file Table_1.docx]

**Table S1. Baseline Characteristics by Status from 2008/2009 to 2011/2012**

| **Characteristics** | **Surviving**  **7342 (54.9)** | **Died**  **3920 (29.3)** | **Lost in Follow-up**  **2102 (15.7)** | ***P*** |
| --- | --- | --- | --- | --- |
| **Sociodemographic** |  |  |  |  |
| Age (years) ^*^ | 81.9 (10.5) | 92.1 (8.8) | 87.0 (11.4) | **<0.001** |
| Gender (female) | 3924 (53.5) | 2213 (56.5) | 1192 (56.7) | **0.002** |
| Race (minority) | 473 (6.4) | 330 (8.4) | 72 (3.4) | **<0.001** |
| Marital status (SDW) | 4127 (56.2) | 3158 (80.6) | 1503 (71.5) | **<0.001** |
| Residence (rural) | 4500 (61.3) | 2578 (65.8) | 905 (43.1) | **<0.001** |
| Occupation (professional) | 604 (8.2) | 197 (5.0) | 211 (10.1) | **<0.001** |
| Education (≥ 1 year) | 3304 (45.1) | 1190 (30.4) | 872 (41.7) | **<0.001** |
| BMI (kg/m^2^) ^*^ | 20.8 (3.5) | 19.6 (3.4) | 20.6 (3.4) | **<0.001** |
| Current smoker | 1515 (20.6) | 620 (15.8) | 292 (13.9) | **<0.001** |
| Current alcohol drinker | 1474 (20.1) | 648 (16.5) | 274 (13.0) | **<0.001** |
| Prefer living alone | 3558 (47.1) | 1199 (29.2) |  | **<0.001** |
| Living arrangements |  |  |  | **<0.001** |
| LWC | 4151 (56.5) | 2897 (73.9) | 1366 (65.0) |  |
| LWS | 1986 (27.1) | 445 (11.4) | 376 (17.9) |  |
| LA | 1205 (16.4) | 578 (14.7) | 360 (17.1) |  |
| **Socioeconomic status** |  |  |  |  |
| Sufficient financial support | 5773 (78.6) | 3056 (78.0) | 1735 (82.5) | **<0.001** |
| Economic independence | 2413 (32.9) | 560 (14.3) | 713 (33.9) | **<0.001** |
| Adequate medical service | 6905 (94.1) | 3608 (92.0) | 1950 (92.8) | **<0.001** |
| Public medical payment | 943 (12.8) | 388 (9.9) | 473 (22.5) | **<0.001** |
| **Dietary habits** |  |  |  |  |
| Fruit eating | 2958 (40.3) | 1380 (35.2) | 1002 (47.7) | **<0.001** |
| Vegetable eating | 6677 (91.0) | 3357 (85.6) | 1857 (88.3) | **<0.001** |
| Tea drinking | 3167 (43.2) | 1446 (36.9) | 916 (43.6) | **<0.001** |
| **Physical and cognitive health status** | |  |  |  |
| Social/leisure activity score (point) ^*^ | 4.2 (3.1) | 2.3 (2.6) | 3.5 (3.2) | **<0.001** |
| Physical exercise | 2523 (34.4) | 807 (20.6) | 677 (32.2) | **<0.001** |
| Poor self-reported health | 1030 (14.0) | 757 (19.3) | 303 (14.4) | **<0.001** |
| Poor interviewer-rated health | 691 (9.4) | 834 (21.3) | 320 (15.2) | **<0.001** |
| Comorbidities (≥ 2) | 3416 (46.5) | 1707 (43.6) | 1055 (50.2) | **<0.001** |
| Serious illness in the past 2 years | 1158 (15.8) | 670 (17.1) | 373 (17.8) | **0.045** |
| Hearing problem | 715 (9.7) | 1053 (26.9) | 425 (20.2) | **<0.001** |
| Visual impairment | 787 (10.7) | 924 (23.6) | 414 (19.7) | **<0.001** |
| Frailty | 917 (12.5) | 1611 (41.1) | 613 (29.2) | **<0.001** |
| Cognitive impairment | 795 (10.9) | 1277 (32.6) | 460 (22.0) | **<0.001** |
| Loneliness | 2019 (27.5) | 1542 (39.3) | 806 (38.3) | **<0.001** |

*Note.* SDW, Single/Separated/Divorced/Widowed; LWC, living with children mainly; LWS, living with spouse only; LA, living alone. ^*^ Kruskall-Wallis test was used, data presented as mean (SD); for other characteristics, χ2 test was used and data presented as n (%).

**Table S2. The Associations of Living Preferences and Living Arrangements with Stratified Status of Loneliness** **Compared with Never Lonely**

| **Cross-sectional analyses** | **OR (95% CI)** | ***P*** |  | **OR (95% CI)** | ***P*** |
| --- | --- | --- | --- | --- | --- |
|  | **Seldom Lonely** | |  | **Sometimes Lonely** | |
| **Living arrangements** |  |  |  |  |  |
| LWC | 1.00 |  |  | 1.00 |  |
| LWS | 0.85 (0.74-0.98) | **0.024** |  | 0.62 (0.52-0.73) | **<0.001** |
| LA | 1.14 (0.97-1.35) | 0.107 |  | 2.22 (1.89-2.60) | **<0.001** |
| **Living preferences** |  |  |  |  |  |
| PreLWC | 1.00 |  |  | 1.00 |  |
| PreLA | 0.78 (0.69-0.88) | **<0.001** |  | 0.75 (0.66-0.86) | **<0.001** |
|  |  |  |  |  |  |
| PreLWC and LWC | 1.00 |  |  | 1.00 |  |
| PreLWC but LWS | 0.90 (0.70-1.15) | 0.400 |  | 0.77 (0.57-1.02) | 0.071 |
| PreLWC but LA | 1.09 (0.79-1.51) | 0.585 |  | 2.26 (1.68-3.03) | **<0.001** |
| PreLA but LWC | 0.78 (0.67-0.91) | **0.001** |  | 0.79 (0.67-0.93) | **0.004** |
| PreLA and LWS | 0.67 (0.58-0.76) | **<0.001** |  | 0.45 (0.38-0.53) | **<0.001** |
| PreLA and LA | 0.91 (0.77-1.07) | 0.272 |  | 1.70 (1.45-1.99) | **<0.001** |
|  | **Often Lonely** | |  | **Always Lonely** | |
| **Living arrangements** |  |  |  |  |  |
| LWC | 1.00 |  |  | 1.00 |  |
| LWS | 0.46 (0.34-0.63) | **<0.001** |  | 0.69 (0.42-1.15) | 0.156 |
| LA | 3.72 (2.95-4.69) | **<0.001** |  | 5.13 (3.50-7.51) | **<0.001** |
| **Living preferences** |  |  |  |  |  |
| PreLWC | 1.00 |  |  | 1.00 |  |
| PreLA | 0.83 (0.68-1.03) | 0.101 |  | 0.76 (0.53-1.08) | 0.129 |
|  |  |  |  |  |  |
| PreLWC and LWC | 1.00 |  |  | 1.00 |  |
| PreLWC but LWS | 0.62 (0.37-1.06) | 0.080 |  | 1.17 (0.53-2.57) | 0.696 |
| PreLWC but LA | 5.65 (3.95-8.08) | **<0.001** |  | 10.2 (6.24-16.8) | **<0.001** |
| PreLA but LWC | 1.19 (0.91-1.55) | 0.194 |  | 1.66 (1.05-2.61) | **0.029** |
| PreLA and LWS | 0.41 (0.30-0.56) | **<0.001** |  | 0.59 (0.34-1.02) | 0.059 |
| PreLA and LA | 2.98 (2.36-3.76) | **<0.001** |  | 3.70 (2.48-5.52) | **<0.001** |

*Note.* LWC, living with children mainly; LWS, living with spouse only; LA, living alone; PreLWC, preferring living with children; PreLA, preferring living alone/only with spouse.

Adjusted for age, gender, race, residence, occupation, education, BMI, smoking, alcohol drinking, living arrangement, living preference, socioeconomic status, dietary habits, social/leisure activity score, physical exercise, poor self-rated health, poor interviewer-rated health, comorbidities (≥2), serious illness in the past 2 years, hearing problem, visual impairment, cognitive impairment, and frailty.

Longitudinal analyses were not done because of the small sample size for incident loneliness.
